# St. George's Hospital

**Published:** 1832-03

**Authors:** 


### ST. GEORGE'S HOSPITAL.

*Clinical Lecture, delivered by* Mr. Brodie, *on a Case of Diseased Knee, complicated with other Affections. With Remarks on the Ligature of Arteries, and Secondary Hemorrhage.**

I shall occupy your attention today by making some remarks on the case of Sarah Hornby. It is one of much practical interest and importance, on account of the great obscurity of the symptoms, which have frequently led me to form a wrong diagnosis: indeed, even the very last opinion which I gave on the case I now find was a false one.

The following is its history: She was admitted on the 17th of November, 1830, under the care of Dr. Hewitt, labouring under

* The Lancet.

dyspepsia and pyrosis, and other symptoms of functional derangement of the stomach and liver. Symptoms of gastritis shortly after appeared, and a blister and leeches were applied for their relief. Five days afterwards she was seized with severe rigors, accompanied by vomiting and pains in the head. On the succeeding day, a pale blush of erysipelas was spread over the chest and breasts. On the 25th, the erysipelas over the surface of the chest appeared to be diminishing, but it extended to the back; and she now complained, for the first time, of pain in the left knee, which became red and swelled, and a slight effusion into the cellular texture beneath could be detected by the touch. On this day also the presence of aphthous scales was discovered in the interior of the mouth. There was also diarrhœa, accompanied by great irritability of the stomach and bowels, paleness and anxiety of the countenance.

Here then, gentlemen, you will perceive there was extensive swelling, inflammation, and effusion under the skin, in the immediate neighbourhood of an important joint. The constitutional disturbance was very great, and the digestive organs were evidently, from the presence of aphthæ, greatly deranged. On the 28th, the knee was still very painful; the inflammation had increased; the leg and thigh were swollen and œdematous, with a very pale tinge of erysipelas suffused over the whole; the upper third of the thigh was tender upon pressure. Leeches were applied, which gave relief. On the 6th of December, there was redness over the patella, heat of the surface of the knee, and a diffused redness and swelling over the lower part of the thigh. Leeches were again applied with advantage; and, on December 9th, the redness was much fainter and more circumscribed, being confined to the neighbourhood of the patella. Dec. 11th, the Linimentum Camphoræ was applied to the knee, and gave no pain. Dec. 14th, a circumscribed purpleness of the skin appeared on the outer and inferior part of the knee. The inferior third of the thigh was much swollen and distended, indicating effusion into the subcutaneous cellular texture external to the joint. The patella bore pressure without pain, but some uneasiness was complained of when pressure was made above the inner condyle of the femur.

On the 9th of January a tumor was discovered, presenting itself on the axillary margin of the left pectoralis major muscle. It was on this day that I first saw her. On the 20th, the abscess was opened, and two ounces of pus were discharged. During all this period the knee remained much in the same state. On the 1st of March an abscess presented itself on the anterior and outer surface of the patella: it was opened, and pus, mixed with the synovia of the joint, escaped; the abscess in the axilla still continued to discharge. March 12th, another abscess presented itself just above the inner condyle, which was opened, and sixteen ounces of pus were discharged. 24th: The orifice of the last abscess has closed. A violent paroxysm of pain and rigors came on in the night; fluctuation above the knee could now be distinctly felt. April 20th, a seton was made in the neighbourhood of the thigh, near the affected parts. Dr. Hewitt on this day discontinued his attendance, and the case was transferred to me.

Here then were various important symptoms presenting themselves, and demanding a very careful and cautious diagnosis in discriminating the real seat of the disease. There was inflammation of the joint, with infiltration of fluid under the cellular texture of the thigh, and there was an extensive abscess formed on the side of the chest. When I first saw her, it was very difficult to say where the real seat of the disease was, there were so many complicated symptoms present; and when I made the seton in the thigh, I found very great resistance to the passage of the seton needle, from the parts being so consolidated together from the effusion of lymph. She was now kept quiet in bed for some months; but the wounds, except that in the axilla, continued still to discharge pus. No motion was attempted, and the leg remained œdematous, the œdema being very firm in its consistence. Two sinuses existed, the result of the original incisions; one on the outside of the patella, the other over the internal condyle. From this last sinus something like exposed bone could be felt, not however distinctly, and I now find that there was no dead bone.* What I felt, therefore, must have been healthy bone covered with periosteum.

Her constitution was too weak to bear such protracted suffering, and, after a period of more than a twelvemonth from the commencement of the disease, her health sank: there was a great discharge of pus from the two incisions; the leg and thigh were œdematous. As there was no other prospect of cure, I determined on amputating the limb above the knee, and the operation was accordingly performed on 19th January, 1832, fourteen months from the commencement of the disease.

*The Operation.* Well, what were the steps of it? A circular incision was first made through the skin and cellular membrane. I had first to cut to a great depth, on account of the large quantity of fat, and the parts beneath being of a very consolidated texture, owing to the inflammation and infiltration of the cellular texture. So much was this the case, that I was not able to retract the skin, but was obliged to dissect it up to some extent. In general there is no trouble in retracting the skin. Your first incision cuts through the fat, adeps, and fascia, and the muscles retract easily, as the fascia has, in general, a very slight connexion with them. Here, however, the fascia seems lost in the mass of diseased structures, which were all infiltrated with lymph, and I was obliged to dissect it up, and separate it from the muscles, which were fat and flabby. There was more venous than arterial hemorrhage during the operation. I drew out the femoral artery with the tenaculum, and tied it; but, owing to the peculiar circumstances of the case, I

* Mr. Brodie had a section of the diseased parts before him.

was obliged to secure the other arteries of the limb with a needle and a ligature, as the lymph bound them closely to the neighbouring parts.

*Ligature of Arteries.* Now it is very rarely that I do take up arteries in this manner, and only upon very extreme and urgent occasions. I would not by any means recommend you to do it. When I was a student, this plan of securing bleeding vessels by means of a needle and ligature was always adopted, and it was not until after the experiments made by Dr. Jones that the method was proved to be a bad one. But how do you proceed to take up an artery with the needle and ligature? Why, you put the needle through the flesh on one side of the artery, and then through the flesh on the other side of the artery, and tie the two ends of the ligature together, and thus you secure the vessel. But by this method you include with the vessel a great portion of the surrounding parts in the ligature; consequently it does not make sufficient pressure on the vessel to cut through its inner coat: and this is frequently a cause of secondary hemorrhage occurring after amputations. But of this I shall say more to you presently. Before Dr. Jones's experiments were published to the world, surgeons were afraid of drawing a ligature *tightly* around an artery, for fear of cutting through all the coats; or even of simply tying an artery with a ligature, lest the pulsations should throw it off: but, in the present advanced state of surgical knowledge, we know this to be impossible. The more substance you include within the ligature, the more likely you are to have secondary hemorrhage, as you only unite the two inner surfaces of the artery by pressure, and do not cut through the inner coat, which you should always do before tightening your ligature. When Mr. Hunter first performed the operation for popliteal aneurism in this country, he dissected up the artery, and tied it. On the continent they then included the surrounding parts with the artery in the ligature.

After securing all the bleeding vessels, I placed a piece of dry lint between the edges of the wound over the stump, put on a bandage and compress, and sent the patient to bed. Now you will ask me, why I put lint over the wound? I did it to prevent union by the first intention. In all cases where you have abscesses burrowing up between the muscles, or coagulable lymph infiltrated into the cellular texture of a limb, or whenever you have to cut through diseased parts, you will never have union by the first intention. Here the parts were very stiff, and the ends of the muscles would not cover the bone. Had I brought them together, abscesses would have formed, and putrid matter would have been discharged, from air being pent up among the rigid muscles. When healthy parts are divided, and you wish to unite them by the first intention, you must not be contented with simply bringing the cut surfaces into contact, but you must take great pains to exclude the air, and all extraneous substances whatever, from the wound, and it is only by this method that you will ever secure union by the first intention.

Secondary hemorrhage, after eight days, came on in this case. Now, secondary hemorrhage may come on a few hours after an operation, after the faintness has gone off, or from the ligature having been put on carelessly. If secondary hemorrhage do not come on in eight or ten hours, inflammation is set up, and then for a time there is no chance of hemorrhage returning; but after some days ulceration comes on, and in those cases where the artery has been taken up with the needle and ligature, and the inner coat is not cut through, this ulceration produces secondary hemorrhage. It is perfectly useless for you to attempt to draw out the bleeding vessel with the tenaculum. You cannot do it; the parts are imbedded in lymph. Then what are you to do? You must remove all the dressings, open the stump, and expose the bleeding surface to the air, and in general the hemorrhage will cease: if it do not, you must make pressure higher up in the course of the artery. This method proved successful in the present case: but even with these means the hemorrhage may occur every second day, and if you do nothing, the patient will die. Styptics seldom do any good. Caustic is better, but it is a barbarous remedy. The only thing you can do, is to operate for aneurism. If secondary hemorrhage should occur in this case, I shall cut down on the femoral artery, below the profunda, and tie it; and this is generally the best method for stopping secondary hemorrhage from the lower extremity. I was once called to a case of secondary hemorrhage from a wound of the palmar arch, and I cut down upon the humeral artery, and tied it; and I really think this is a better plan than performing two operations for securing the ulnar and radial arteries.

*Examination of the Joint. Remarks:* On a dissection being made of the joint, no dead bone was discovered: I was, therefore, wrong in my diagnosis, but portions of the cartilage were here and there absorbed, and complete anchylosis had taken place. Now, I think that the original disease was in the synovial membrane of the joint, and extended thence to the superficial cellular texture, where it was more violent, and terminated in the effusion of pus. Similar symptoms to these occurred in a patient who had erysipelas, in this hospital, some years ago.

Mucous membranes suppurate more readily than serous ones. The pus here was secreted by the synovial membrane, and the bones bore but slight marks of disease. In grown-up persons, suppuration and anchylosis (when the latter does occur,) are seldom cured. Anchylosis is more common in grown-up persons than in adults.

In this case there were abscesses about the joints, which did not heal. If they had healed, she might have recovered; but, with an anchylosed joint, the abscesses did not heal, because they were pent up between muscles and tendons. The first object which you should have in view towards healing an abscess, should be to take care that the pus has a free exit. In the knee, the leg, and the thigh, abscesses are situated among muscles. Abscesses do not heal so readily among unhealthy parts as among parts that are healthy: here the parts were unhealthy, and the abscesses did not therefore heal.

### CRITICAL ANALYSES.

